# Association of relative telomere length with progression of chronic kidney disease in two cohorts: effect modification by smoking and diabetes

**DOI:** 10.1038/srep11887

**Published:** 2015-07-07

**Authors:** Julia Raschenberger, Barbara Kollerits, James Ritchie, Beverley Lane, Philip A. Kalra, Eberhard Ritz, Florian Kronenberg

**Affiliations:** 1 Division of Genetic Epidemiology, Department of Medical Genetics, Molecular and Clinical Pharmacology, Medical University of Innsbruck, Innsbruck, Austria; 2 Vascular Research Group, The University of Manchester Academic Health Science Centre, Salford Royal NHS Foundation Trust, Salford, United Kingdom; 3 Department of Internal Medicine, Division of Nephrology, Ruprecht-Karls-University, Heidelberg, Germany

## Abstract

Chronic kidney disease (CKD) is a highly progressive disease. We studied the association between relative telomere length (RTL) and CKD progression and tested whether this association is modified by smoking and diabetes mellitus. RTL was measured by qPCR in two prospective cohort studies, the MMKD-Study (n = 166) and the CRISIS-Study (n = 889) with a median follow-up of 4.5 and 2.8 years, respectively. Progression was defined as doubling of baseline serum creatinine (MMKD-Study) and/or end stage renal disease (both studies). 59 and 105 of the patients from MMKD and CRISIS experienced a progression of CKD. Mean standardized pooled RTL was 0.74 ± 0.29. In the meta-analysis shorter RTL at baseline showed a borderline association with CKD progression (HR = 1.07 [95%CI 1.00–1.15]; p = 0.06). We observed an effect modification of RTL and CKD progression by smoking and diabetes (p-values of interaction p = 0.02 and p = 0.09, respectively). Each 0.1 unit shorter RTL was significantly associated with an increased hazard for CKD progression in active-smokers by 44% (HR = 1.44 [1.16–1.81]; p = 0.001) and in patients with diabetes mellitus by 16% (HR = 1.16 [1.01–1.34]; p = 0.03). Estimates were adjusted for baseline age, sex, proteinuria and GFR. This study in two independent cohorts reinforces that RTL is a marker and potentially a pathogenetic factor for CKD progression.

Chronic kidney disease (CKD) represents with roughly 11% a highly prevalent and life-threatening disease and this frequency increases steadily^1^,^2^. A significant number of patients with CKD are at risk of progressive loss of renal function. Functional loss of the kidney is not only attributable to age, but also to risk factors such as smoking and diabetes mellitus^3^,^4^. Although several risk markers for CKD progression have been identified to date^5^, the underlying mechanisms and the prediction of progression have not been fully elucidated. Further risk factors and markers are therefore of great interest.

Telomeres are regions of random repetitive nucleotide sequences (5–15 kb) at the end of eukaryotic chromosomes. Their principle task is to sustain chromosomal integrity^6^. With aging process, DNA polymerase cannot completely replicate the 3’-end of the linear DNA for lack of the required RNA primer at this position. This results in a loss of telomere repeats with each cell division (‘end-replication-problem‘^7^). When the telomere length (TL) has become critically short (Hayflick limit^8^), cellular senescence or apoptosis occur^9^. This ends in cell cycle G_1_ arrest at advanced age causing reduced proliferation, resulting in less efficient regeneration and repair of tissue including the kidney^10^.

Additionally, telomeres of somatic cells shorten as a result of oxidative stress^11^ and inflammation^12^ once telomerase or alternative-lengthening mechanisms are not operative^13^. A deregulated renin-angiotensin system can decrease TL due to oxidative stress and inflammation^14^. In addition the risk of CKD is negatively influenced by an impaired immunity^10^,^15^, a known predictor of morbidity and mortality in the elderly. Well known risk factors such as smoking are reported to be associated with short TL^16^,^17^. Decreased TL is also observed in the presence of several age-related diseases. Results from the prospective Bruneck Study^18^ and a meta-analysis additionally including the two prospective studies Strong Heart Family Study^19^ and Women’s Health Initiative^20^ revealed a clear association between low relative TL and incident type 2 diabetes mellitus^18^. This and other observations have led to the proposal that decreased TL is an indicator of biological age and a potential marker of disease risk and progression^21^,^22^. The potential causal role of telomeres in the pathogenesis of age-related diseases, however, is not entirely understood. Reduced TL has been shown to be associated with diseases such as kidney^10^,^23^,^24^,^25^,^26^,^27^ and cardiovascular disease (CVD)^28^,^29^,^30^,^31^,^32^,^33^. Only one study investigated the association of TL with progression of kidney disease in 132 patients with type 1 diabetes: telomere length independently predicted progression to diabetic nephropathy^23^. So far no information is available for progression of non-diabetic kidney disease.

The aim of the present study was to assess the association between RTL and CKD progression and to test whether this association is modified by smoking and diabetes mellitus. Two prospective cohort studies including 1055 non-dialysis-dependent patients at different stages of CKD were used.

## Results

### Baseline Characteristics of Patients

**Figure 1:**
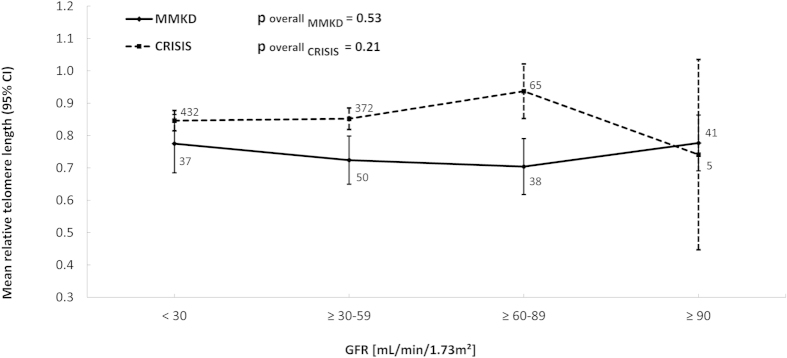
Line plot displaying mean age- and sex-adjusted relative telomere length (RTL) per stages of chronic kidney disease (CKD) defined by the Kidney Disease Outcomes Quality Initiative (KDOQI) guidelines. Error bars refer to the 95% confidence interval (CI). Overall p-values for comparison between GFR groups are obtained from general linear regression models for each study. RTL values of CRISIS and MMKD are not directly comparable as different DNA extraction methods were used in the two studies that have an influence on the measured values^39^. However, each of the studies can be interpreted on its own. Numbers near the lines represent the number of patients in the respective chronic kidney disease stages.

Table 1 provides baseline clinical characteristics and laboratory data of 166 non-dialysis-dependent patients of the MMKD Study and of 889 patients of the CRISIS Study in whom RTL was measured at baseline and who have completed follow-up. Mean ± SD RTL was 0.74 ± 0.27 in the MMKD Study and 0.86 ± 0.34 in the CRISIS Study with a mean standardized pooled RTL of 0.74 ± 0.29. We found a significant correlation between age and RTL in both studies (r = −0.199, p = 0.01 in the MMKD Study, and r = −0.174, p < 0.001 in CRISIS). Mean age- and sex-adjusted RTL was not significantly different across stages of CKD in the MMKD and CRISIS Study (Fig. 1). This holds true even after stratifying for smoking or diabetes status.

### RTL and Progression of CKD

Patients were followed prospectively until the end of the study or occurrence of the primary renal endpoint. Progression was defined as doubling of baseline serum creatinine (MMKD-Study) and/or end stage renal disease (both studies). Median follow-up time in the MMKD Study was 4.5 years (interquartile range 2.7–5.2) and in CRISIS 2.8 years (interquartile range 1.3–4.1), respectively. Of the 166 MMKD patients, 59 patients (35.5%) reached the endpoint defined as doubling of baseline serum creatinine and/or end-stage-renal-disease necessitating renal replacement therapy. Of the 889 CRISIS patients, 105 patients (11.8%) reached the endpoint of end-stage-renal-disease necessitating renal replacement therapy. RTL were slightly, but not significantly shorter in the patients reaching the endpoint as compared to those without CKD progression (MMKD: 0.70 ± 0.24 versus 0.77 ± 0.29, p = 0.11; CRISIS: 0.80 ± 0.26 versus 0.86 ± 0.35, p = 0.26). In CRISIS a significantly greater proportion of patients with CKD progression suffered from diabetes mellitus compared to those without progression (42% vs. 32%, p = 0.039). Further characteristics of the patients with and without CKD progression are presented in Table 1. In both studies, patients with CKD progression had a lower GFR as well as a higher proteinuria at baseline. There was no significant difference in mean age-adjusted RTL between men and women.

Table 2 presents the results of multiple Cox regression analyses for both studies and for the meta-analysis. In the meta-analysis, shorter RTL at baseline was associated with higher risk of CKD progression (HR = 1.07 [95%CI 1.00–1.15], p = 0.06; adjusted for age, sex, proteinuria and GFR). We found a clear and significant interaction between RTL and smoking status in both studies (each p = 0.02). In the MMKD Study the age-, sex-, proteinuria- and GFR-adjusted HR to reach a progression endpoint was significantly higher by 35% with each decrement of 0.1 unit of RTL in active-smokers (HR = 1.35 [95%CI 1.01–1.79], p = 0.04). Such risk increase for RTL was not observed in non- and ex-smokers (HR = 1.07 [95%CI 0.94–1.21]; p = 0.32). The results of the MMKD Study were confirmed by the CRISIS Study: for each decrement of 0.1 unit of RTL the HR for progression of CKD was increased by 61% for active-smokers (HR = 1.61 [95%CI 1.13–2.30]; p = 0.009) whereas it was unchanged for non- and ex-smokers (HR = 1.01 [95%CI 0.91–1.11]; p = 0.92). The meta-analysed results revealed a significantly increased risk for active-smokers (HR = 1.44 [95%CI 1.16–1.81]; p = 0.001) but not for non- and ex-smokers (HR = 1.03 [95%CI 0.95–1.11]; p = 0.49). In addition, we investigated the yearly GFR change in patients from the CRISIS Study. Active smokers in the tertile with the highest GFR change had significantly lower RTL at baseline compared the those in the tertiles with the lowest and middle GFR changes (mean ± SEM: 0.68 ± 0.05 versus 0.84 ± 0.06 and 0.94 ± 0.06, p = 0.047 and p = 0.002, respectively; data are adjusted for age, sex, proteinuria and GFR at baseline). No significant differences were observed in the combined group of non-smokers and ex-smokers.

Furthermore, we found an indication for an interaction between RTL and diabetes status in CRISIS (p = 0.09). Shorter RTL predicted faster progression of CKD in patients with diabetes mellitus (HR = 1.16 [95%CI 1.01–1.34]; p = 0.03), but not in patients without diabetes mellitus (HR = 0.96 [95%CI 0.86–1.07]; p = 0.47). This issue could not be investigated in the MMKD Study since that study has included only non-diabetic patients. When we analysed the yearly GFR change in relation to RTL at baseline we could not observe significant differences in mean RTL between tertiles of GFR change in diabetic and non-diabetic patients.

### Sensitivity analysis

Hypertension was present in 94% and 88% of the patients in the CRISIS and MMKD Study. In both studies more than 90% received antihypertensive medications. In a sensitivity analysis we adjusted the meta-analysis (Cox regression model 3) in active-smokers additionally for angiotensin-converting-enzyme inhibitors. The effect estimates of RTL remained nearly unchanged. The same holds true for patients with diabetes and when adjusting model 3 for angiotensin receptor blockers (ARBs) (available in CRISIS only) (for details see Supplementary Material).

Additionally, we adjusted Cox regression model 3 for cardiovascular disease at baseline. These analyses revealed no difference in risk estimates of RTL.

## Discussion

To our knowledge, this is the first study in patients with mild to severe CKD that shows a significant effect modification by smoking and diabetes on the association of RTL measured in DNA from blood leukocytes with CKD progression. This association between short RTL and progression was only observed in active-smokers and patients with diabetes mellitus. The observed effects in both cohorts were independent of age, sex, proteinuria and GFR measured at baseline.

This study has two central questions 1) the association of TL with kidney function at baseline in a cross-sectional analysis and 2) the association of baseline TL with CKD progression during the prospective follow-up.

For the first issue, it is impossible to differentiate which comes first, kidney impairment followed by shortening of TL or vice versa. The finding of an association in one or the other direction, or finding no association could be markedly influenced by confounding, e.g. biological processes resulting in an elongation of TL caused by an activation of the telomere-lengthening enzyme telomerase. Evidence is accumulating that telomere-lengthening is not as rare as previously thought. Data indicate that the TL pattern is oscillating and levelling out over longer follow-up^34^. A community-based prospective cohort of 5297 participants reported that after a median follow-up of 6.6 years RTL either shortened (44% of subjects), remained stable (22%) or even elongated (34%)^35^.

For the second question, if RTL predicts CKD progression, we can only speculate whether RTL is a risk marker or risk factor for CKD progression. Our observation that the association of RTL with CKD progression is strongest in active-smokers and patients with diabetes mellitus supports the concept that telomere shortening is markedly influenced by oxidative stress and inflammation. Both groups are known to be more exposed to these processes. This would indicate that RTL may not reflect kidney function per se, but rather the kidney function influenced by environmental factors. In line with this, we found no significant differences of age- and sex-adjusted mean RTL across GFR stages in the entire group as well after stratifying by smoking or diabetes mellitus in both cohorts. Patients from the CRISIS Study had a greater degree of renal impairment at baseline compared to patients in the MMKD Study (34 ± 17 versus 64 ± 40 mL/min/1.73 m^2^). Nevertheless, the association of RTL with CKD progression was seen in both studies, supporting the hypothesis that RTL reflects the environmental-impact on kidney function, even in earliest stages of renal impairment.

A deregulated renin-angiotensin-aldosterone system (RAAS) was described to contribute to TL shortening and renal fibrosis as a result of oxidative stress and inflammation^14^. It is well known that angiotensin II activates NAD(P)H oxidase to produce reactive oxygen species resulting in oxidative stress and kidney damage. It is a powerful activator of the cellular senescence p53/p21 pathways and causes increased blood pressure and vasoconstriction, thus promoting vascular senescence. This concept is in line with the finding in other age-related diseases that decreased TL^10^,^23^,^24^,^28^,^32^ influenced by the above factors. Therefore TL is believed to be an indicator of biological age and a potential marker of disease risk and progression.

Persistent inflammation and oxidative stress may affect RTL independently of chronological age in CKD patients. Oberg *et al.* compared a small cohort of 60 patients with stage 3–5 CKD not receiving renal replacement therapy with healthy subjects and showed that several biomarkers of inflammation and oxidative stress differed significantly. Higher levels of these biomarkers were found in patients with diabetes mellitus. There was, however, no close relationship between eGFR and any of these biomarkers, indicating that increased oxidative stress and inflammation exert their influence even before terminal renal failure is reached^36^. Accordingly, Verzola *et al.* reported that yet in the absence of renal failure cellular senescence may be present^37^.

Hyperglycemia triggers generation of free radicals and oxidative stress. Either directly or indirectly, it also exhausts the replicative capacity in selected kidney cell populations causing loss of the ability to replicate and repair^37^. Finally, diabetes mellitus affects pathways responsible for hyperglycemia-induced cell damage that is linked to accelerated telomere shortening^38^. This finding fits to our observation that reduced RTL is particularly predictive for CKD progression in patients with diabetes mellitus.

The risk of CKD is increased by impaired immunity, a predictor of morbidity and mortality specifically in the elderly. Immune cells are highly proliferative. Therefore it is important to maintain TL to ensure stable cell replication. It has even been suggested that TL might be a limiting factor for cell survival and consequently for tissue and the entire kidney. It is known that nephrons loose functionality at some point of CKD. One of the potential triggers might be TL. Which conditions actually influence this time point is not clear. We suspect that TL might be the promising link. This potential pathogenic link is not proven by our study, but it is an attractive speculation that it might be the sum of oxidative-stress-mediated damage and replication of immune cells to ensure renal repair and regeneration.

Only one recent study in 132 patients with type 1 diabetes investigated the role of RTL on diabetic nephropathy progression^23^. The major strength of our study is that we investigated the relationship between TL and CKD progression in two independent cohorts with mild to severe CKD with an *a priori* defined analysis plan. Although two different renal endpoint definitions and DNA extraction methods were applied, we identified the same direction of association of RTL with CKD progression.

It might be considered a limitation of the study that we saw major differences in RTL between the two studies: longer telomeres were observed in the CRISIS Study whose participants were on average older and had a lower GFR than those of the MMKD Study. This is at the first glance counterintuitive but is explained by the fact that T/S-ratio values are not fully comparable between the two studies, since different DNA extraction methods were used. It was recently demonstrated that DNA extraction methods have a systematic influence on the TL measurement^39^ (and manuscript in preparation). It was not possible to use the same DNA extraction method for both studies, since the DNA had already been extracted at the time RTL was measured. Since the difference between the two DNA extraction methods seems to be of systematic nature and the method was not changed within each single study, the data had to be analyzed for each study separately. However, the effect estimates in both studies pointed in the same direction, which allowed performing a meta-analysis of the two studies.

Our study is limited by its observational design and thus is not able to prove causality. It included mainly Caucasian individuals and needs confirmation in different ethnicities. Another limitation is that DNA was extracted from whole blood and we therefore measured mean leukocyte TL from a mixture of different cell subsets. Knowing TL from various kidney cell types would be of interest but is not possible to obtain in a large epidemiological study. However, investigations in cats revealed shorter TL in animals with compared to those without CKD^40^. In case TL in kidney cells indeed resembles the pathogenic course of the disease in a better way, we might have rather underestimated than overestimated the association between TL measured in DNA from whole blood and progression of CKD. Finally, our analysis is limited by the fact, that we did not have GFR slopes available in both studies and the main analysis included only endpoints such as doubling of serum creatinine and/or ESRD to allow the meta-analysis of the data from both studies. However, to avoid a loss of information by categorization of continuous data, we included in CRISIS as an additional analysis the association of RTL at baseline with yearly GFR changes calculated from the GFR difference between baseline and last follow-up.

In summary, this study in two independent cohorts of patients with mild to severe CKD indicates a significant association of shorter RTL with CKD progression in active-smokers as well as in patients with diabetes mellitus. Our observations reinforce the paradigm that telomere length is a marker presumably reflecting the impact of genetic and environmental risk factors in the pathogenesis of CKD progression.

## Methods

### Mild to Moderate Kidney Disease Study (MMKD Study)

The methodology of the MMKD Study has previously been reported in detail^41^. Briefly, the MMKD Study is a prospective cohort study including 227 adult Caucasian non-diabetic patients at various CKD stages recruited in Germany, Austria, and South Tyrol (Italy) as previously described. The patients had stable kidney function for at least 3 months before inclusion into the study. The glomerular filtration rate (GFR) was assessed in all patients using the iohexol clearance technique as described in detail elsewhere^42^.

The endpoint CKD progression was defined as doubling of baseline serum creatinine and/or terminal renal failure necessitating renal replacement therapy (dialysis therapy or kidney transplantation). This endpoint definition was decided *a priori* in the planning phase of the study in the years 1996/97 and was in line with Maschio *et al.*^43^. This definition was used in all of the previous publications of the MMKD Study (e.g.^41^,^44^,^45^). A total of 177 (78%) patients could be followed prospectively. RTL was available in 166 patients. All data reported in this manuscript are based on these subjects. Further details are provided as Supplementary Material. The examination protocol was approved by the Ethics Committee of the Medical University of Innsbruck in accordance with the Declaration of Helsinki. Written informed consent was obtained from each participant.

### Chronic Renal Insufficiency Standards Implementation (CRISIS Study)

The CRISIS Study is an ongoing prospective non-dialysis-dependent CKD cohort of a predominantly white population in Greater Manchester referred for secondary care management of patients at different CKD stages. The study investigates factors associated with CKD progression, cardiovascular events and mortality. Inclusion criteria were defined as estimated GFR (eGFR) 10–59 ml/min/1.73 m^2^. The study was approved by The Central Manchester Research Ethics Committee in accordance with the Declaration of Helsinki. Written informed consent was obtained from each participant.

Details of data recorded in CRISIS have been published previously^46^. Estimated GFR was calculated according to the CKD-EPI equation. The endpoint of the prospective follow-up period was defined as advanced renal failure necessitating renal replacement therapy (dialysis therapy or kidney transplantation). DNA and follow-up data are available from 889 patients. Reported data are based on these patients. About a third of the patients had diabetes mellitus. In an additional analysis we calculated the yearly GFR change by calculating the GFR difference between baseline and last follow-up divided by the number of years followed (and censored at the time of start of renal replacement therapy).

### Measurement of relative telomere length (RTL)

Genomic DNA from the MMKD Study blood samples was extracted from frozen EDTA-blood samples with the EZ1®DNA Blood 200 μl Kit using the Qiagen EZ1 advanced Biorobot whereas in CRISIS it was extracted with organic extraction with phenol /chloroform /isoamylalcohol. RTL in both studies was measured in a single laboratory using a quantitative polymerase chain reaction (qPCR) approach developed by Cawthon^47^ to measure the T/S-ratios. The protocol was modified with regard to control samples and data processing with modifications described recently^33^. The T/S-ratios are proportional to individual RTL. Briefly, each qPCR plate contained the standard DNA, a quality control (commercially available DNA-Human Genomic DNA, Roche) and a non-template control. All samples, standards and controls were analyzed in quadruplicate. After each qPCR run a melting curve analysis was performed to verify the specificity and identity of the products.

Several methods of TL measurement have been published so far. The comparison between these two methods used within the project at hand has been published previously^32^.

Further details are provided as Supplementary Material.

### Statistical Analysis

Continuous variables were compared between groups using unpaired t-tests or the Wilcoxon rank-sum test where appropriate. Dichotomized variables were compared using Pearson χ^2^-test. We explored the correlation of RTL with age using Spearman correlation analysis. A line plot displaying mean age- and sex-adjusted RTL per stage of CKD defined by the Kidney Disease Outcomes Quality Initiative (KDOQI) guidelines was undertaken. Univariate and adjusted Cox proportional hazard regression analyses were applied to calculate hazard ratios (HRs) and corresponding 95% confidence intervals per decrement of 0.1 unit change of RTL for the progression to renal endpoints. The proportional hazards assumption was tested. Using non-linear splines in both studies, the effect of RTL did not depart from linearity in the fully adjusted model 3.

We also tested the hypothesis that the association of RTL with CKD progression is modified by factors such as smoking or diabetes mellitus. Interaction between these parameters and RTL was tested by integrating multiplicative interaction terms in the Cox proportional hazards models. A p value < 0.10 for interaction terms was considered as significant. A pooled effect size for the respective studies was calculated using a fixed effects meta-analysis as there was no indication for violation of the assumption of heterogeneity. Furthermore, marginal mean values and SEM of baseline RTL (adjusted for age, sex, proteinuria and baseline GFR) were calculated per tertile of yearly GFR change stratified for smoking status and diabetes diagnosis. This analysis was performed using tertiles since linearity of the data was not given as investigated by a non-linear P-spline in a general linear regression analysis on RTL in active smokers. Yearly GFR change was inverse-normal transformed due to its skewed distribution. For detailed information see Supplementary Material and Supplementary Figure 1.

In all analyses, a p value < 0.05 was considered statistically significant except for interaction terms where we viewed p < 0.10 as statistically significant. Analyses were performed using SPSS for Windows version 21 and R 3.0.1. Further details are provided as Supplementary Material.

## Additional Information

**How to cite this article**: Raschenberger, J. *et al.* Association of relative telomere length with progression of chronic kidney disease in two cohorts: effect modification by smoking and diabetes. *Sci. Rep.* **5**, 11887; doi: 10.1038/srep11887 (2015).

## Supplementary Material

Supplementary Information

## Figures and Tables

**Table 1:** Baseline clinical and laboratory data of 889 patients of the CRISIS Study and 166 patients of the MMKD Study who completed follow-up and stratified by patient groups with and without progression of chronic kidney disease.

**Variable**	**CRISIS**				**MMKD**			
	**All patients (n = 889)**	**Non-progressors (n = 784)**	**Progressors (n = 105)**	**P-value**^a^	**All patients (n = 166)**	**Non-progressors (n = 107)**	**Progressors (n = 59)**	**P-value**^a^
Age (years)	64.0 ± 14.3[56.0;66.0;75.0]	64.6 ± 14.2[57.0;67.0;75.0]	59.5 ± 13.5[56.0;66.0;75.0]	<0.001	46.3 ± 12.2[36.0;49.0;56.0]	44.5 ± 12.6[34.0;48.0;56.0]	49.4 ± 10.8[45.0;51.0;57.0]	0.02
Sex: males/females, n (%)	545/344(61.3/38.7)	478/306(60.9/39.1)	67/38(63.8/36.2)	0.58	109/57(65.7/34.3)	70/37(65.4/34.6)	39/20(66.1/33.9)	0.93
BMI (kg/m^2^)	28.7 ± 5.7	28.9 ± 5.7	27.9 ± 5.3	0.11	25.1 ± 3.8	24.9 ± 3.6	25.7 ± 4.1	0.18
Current smokers, n (%)	100 (11.3)	83 (10.6)	17 (16.2)	0.09	31 (18.7)	16 (15.0)	15 (25.4)	0.15
Diabetes mellitus, n (%)	293 (33.0)	249 (31.8)	44 (41.9)	0.04	NA	NA	NA	NA
Systolic bloodpressure (mmHg)	136 ± 21[120,134,149]	135 ± 20[120,134,147]	140 ± 21[127,139,150]	0.01	136 ± 20	136 ± 22	137 ± 17	0.62
Diastolic bloodpressure (mmHg)	71 ± 11[62,71,78]	71 ± 10[62,71,78]	72 ± 12[62,72,80]	0.55	87 ± 13	86 ± 14	88 ± 12	0.42
High sensitivityC-reactive protein(mg/dL)	0.73 ± 1.26[0.16;0.35;0.81]	0.74 ± 1.26[0.16;0.34;0.84]	0.65 ± 1.21[0.19;0.39;0.73]	0.9	0.28 ± 0.29[0.08;0.17;0.40]	0.26 ± 0.28[0.07;0.17;0.39]	0.31 ± 0.32[0.08;0.19;0.46]	0.36
Serum albumin(g/dL)	4.4 ± 0.4[4.2;4.4;4.6]	4.4 ± 0.4[4.2;4.4;4.6]	4.2 ± 0.5[3.9;4.3;4.5]	0.003	4.6 ± 0.4	4.6 ± 0.4	4.5 ± 0.4	0.66
Proteinuria(g/24 h/1.73 m^2^)	0.51 ± 0.94[0.07;0.15;0.46]	0.41 ± 0.78[0.06;0.13;0.39]	1.26 ± 1.52[0.21;0.58;1.70]	<0.001	0.99 ± 0.92[0.20;0.69;1.52]	0.87 ± 0.96[0.14;0.44;1.25]	1.20 ± 0.81[0.61;0.99;1.72]	0.001
GFR(mL/min/1.73 m^2^)	34 ± 17[20,30,44]	36 ± 17[23,32,46]	18 ± 10[11,15,21]	<0.001	64 ± 40	79 ± 38	37 ± 26	<0.001
Creatinine(μmol/L)	204 ± 102[132,178,248]	185 ± 81[126,168,223]	341 ± 134[244,319,425]	<0.001	189 ± 117[104,144,242]	132 ± 62[93,119,158]	291 ± 125[191,289,392]	<0.001
Relative telomerelength^b^	0.86 ± 0.34	0.86 ± 0.35	0.80 ± 0.26	0.26	0.74 ± 0.27	0.77 ± 0.29	0.70 ± 0.24	0.11

GFR denotes glomerular filtration rate measured by iohexol clearance or in CRISIS calculated according to the CKD-EPI equation, proteinuria is given in g/24 h in CRISIS; BMI; body-mass index. Diabetes mellitus includes Type 1 and Type 2. Data are presented as mean ± SD and 25^th^, 50^th^ (median) and 75^th^ percentiles for skewed variables where appropriate.

^a^P value for comparison between progressors and non-progressors. NA, not applicable.

^b^Relative telomere length is not comparable between the two studies since different DNA extraction methods have been applied (see Discussion).

**Table 2:** The association of relative telomere length (RTL) with progression of kidney disease during the observation period using multiple Cox proportional hazards regression models.

**Relative telomere length (0.1 unit decrease)**		**n^c^ Total / Progressors**	**Model 1^a^**		**Model 2^a^**		**Model 3^a^**	
			**HR (95% CI)**	**p**	**HR (95% CI)**	**p**	**HR (95% CI)**	**p**
CRISIS	All patients	889 / 105	1.05 (0.98–1.12)	0.17	1.09 (1.01–1.17)	0.03	1.05 (0.96–1.14)^b^	0.32
MMKD	All patients^d^	166 / 59	1.06 (0.95–1.18)	0.28	1.07 (0.97–1.19)	0.20	1.11 (0.99–1.25)	0.07
**Meta-analysis MMKD + CRISIS**	**All patients**	**1055 / 164**	**1.05 (0.99**–**1.12)**	**0.08**	**1.08 (1.02**–**1.15)**	**0.01**	**1.07 (1.00**–**1.15)**	**0.06**
CRISIS	Active-smokers	100 / 17	1.37 (1.07–1.75)	0.01	1.54 (1.11–2.13)	0.009	1.61 (1.13–2.30)	0.009
MMKD	Active-smokers	31 / 15	1.17 (0.94–1.46)	0.16	1.16 (0.92–1.45)	0.20	1.35 (1.01–1.79)	0.04
**Meta-analysis MMKD + CRISIS**	**Active-smokers**	**131 / 32**	**1.25 (1.07**–**1.48)**	**0.007**	**1.02 (1.06**–**1.53)**	**0.01**	**1.44 (1.16**–**1.81)**	**0.001**
CRISIS	Non- and ex-smokers	789 / 88	1.02 (0.95–1.10)	0.59	1.05 (0.97–1.14)	0.24	1.01 (0.91–1.11)^b^	0.92
MMKD	Non- and ex-smokers	135 / 44	1.02 (0.90–1.16)	0.72	1.03 (0.90–1.17)	0.66	1.07 (0.94–1.21)	0.32
**Meta-analysis MMKD + CRISIS**	**Non- and ex-smokers**	**924 / 132**	**1.02 (0.96**–**1.09)**	**0.52**	**1.04 (0.997**–**1.12)**	**0.22**	**1.03 (0.95**–**1.11)**	**0.49**
CRISIS	Diabetics	293 / 44	1.09 (0.97–1.23)	0.13	1.11 (0.98–1.24)	0.09	1.16 (1.01–1.34)	0.03
CRISIS	Non-diabetics	596 / 61	1.02 (0.93–1.11)	0.67	1.05 (0.95–1.16)	0.32	0.96 (0.86–1.07)^b^	0.47
**Meta-analysis MMKD + CRISIS**	**Non-diabetics**	**762 / 105**	**1.04 (0.97**–**1.11)**	**0.31**	**1.06 (0.99**–**1.14)**	**0.11**	**1.03 (0.95**–**1.11)**	**0.47**

The hazard ratios (HR) and 95% confidence intervals (CI) were determined by univariate and multiple Cox proportional hazards regression analysis and are indicated for each decrement of 0.1 unit of RTL. Further abbreviations and explanations see footnote of Table 1.

The analyses are stratified for patients with and without diabetes as well as active smoking and non-/ex-smoking status.

^a^The estimates in model 1 are adjusted for age and sex, those in model 2 are adjusted for age, sex and proteinuria and those in model 3 are adjusted for age, sex, proteinuria and GFR.

^b^The estimates of these models are adjusted for age, sex, proteinuria, GFR and a covariate that accounts for the time-dependency of GFR.

^c^N in each group refers to the patients with available RTL. It provides the total number of patients included in the analysis as well as the number of patients with progression of CKD.

^d^The MMKD Study only includes non-diabetic patients.
