# Supplementary material for: Association of relative telomere length with progression of chronic kidney disease in two cohorts: effect modification by smoking and diabetes
Source: Sci Rep. 2015 Jul 7;5:11887. doi: 10.1038/srep11887 (PMC4493689; doi:10.1038/srep11887)
Supplement: Supplementary Information [file srep11887-s1.pdf]

Online Supplementary Material to

Association of relative telomere length with progression of chronic kidney disease in two cohorts: effect modification by smoking and diabetes

Julia Raschenberger [1*], Barbara Kollerits [1*], James Ritchie [2], Beverley Lane [2], Philip A Kalra [2], Eberhard Ritz [3], Florian Kronenberg [1]

*These authors contributed equally.

[1] Division of Genetic Epidemiology, Department of Medical Genetics, Molecular and Clinical Pharmacology, Innsbruck Medical University, Innsbruck, Austria

[2] Vascular Research Group, The University of Manchester Academic Health Science Centre, Salford Royal NHS Foundation Trust, Salford, United Kingdom

[3] Department of Internal Medicine, Division of Nephrology, Ruprecht-Karls-University, Heidelberg, Germany

MATERIALS AND METHODS

Mild to Moderate Kidney Disease Study (MMKD Study)

Exclusion criteria were defined as: serum creatinine >6 mg/dL (531 µmol/L), diabetes mellitus of any type, malignancy, liver, thyroid, or infectious diseases, nephrotic syndrome (defined as daily proteinuria >3.5g/1.73m^2), organ transplantation, immunosuppressive treatment, allergy to ionic contrast media, treatment with fish oil or erythropoietin and pregnancy.

All patients were recruited by a single investigator who visited all participating centers to avoid interobserver differences. Information on age, gender, smoking habits, comorbidities and antihypertensive treatment at baseline was recorded by patient interview and confirmed by checking patient records. A clinical examination completed the procedure. Hypertension was defined as BP >140/90 mmHg and/or the use of antihypertensive medication.

Patients received regular reviews in the outpatient ward. Endpoints were assessed by medical record abstraction and reported to the coordinating center.

Unless they had reached the primary endpoint, patients were followed until the end of the whole follow-up time period of 7 years. A total of 177 (78%) patients from the baseline cohort of 227 patients could be followed prospectively. Due to inappropriate or missing laboratory samples (n=11) or missing of follow-up data (n=50), the values of RTL were available in 166 patients.

A total of 50 patients were lost to follow-up. They moved away or were not referred by their physicians for follow-up visits in the renal units. Significantly better renal function at baseline was observed when compared to patients who were not lost to follow-up (i.e., a higher mean GFR [91±44 versus 64±39 ml/min per 1.73 m^2; P <0.01]). Both groups, however, did not differ significantly with respect to age and gender.

Chronic Renal Insufficiency Standards Implementation (CRISIS Study)

Adult patients referred for management of renal disease at Salford Royal NHS Foundation trust (catchment population 1.55 million) were approached for recruitment in an unselected manner.

Patient characteristics are assessed at baseline and annually thereafter by structured face-to-face interviews performed by trained research nurses. Biochemical parameters are analyzed as part of routine clinical monitoring.

Exclusion criteria were renal replacement therapy at time of referral (dialysis or transplantation) and expected patient or renal survival of less than six months.

Assessing relative telomere length (RTL)

Genomic DNA of the MMKD Study was extracted from frozen EDTA-blood samples with the EZ1®DNA Blood 200 µl Kit using the Qiagen EZ1 advanced Biorobot. DNA concentrations were measured with the Tecan NanoQuant infinite M200.

Genomic DNA of the CRISIS Study was extracted from frozen EDTA-blood samples with organic extraction with phenol /chloroform /isoamylalcohol. DNA concentrations were measured with Nanodrop (ND-1000).

Samples were normalized in 96-well microtiter plates. Samples were ascertained in a singleplex, quadruplicate approach to measure the T/S-ratios. These T/S-ratios are proportional to individual relative telomere length (RTL). RTL was measured with some modifications as described below by using a quantitative real-time polymerase chain reaction (qPCR) assay, which was first described by Cawthon [1].

Singleplex PCRs for telomere (T) and housekeeping gene (S) PCRs were identical composed. DNA samples were run in 15µl reactions containing 1x Quantifast ™ SYBR® Green PCR mastermix (Qiagen), 10 ng of DNA, 1µM of telomere primer or 250nm of housekeeping gene 36B4 primer. The primer sequences (5'→3') were:

tel1b CGGTTTGTTTGGGTTTGGGTTTGGGTTTGGGTTTGGGTT;

tel2b GGCTTGCCTTACCCTTACCCTTACCCTTACCCTTACCCT;

36B4u CAGCAAGTGGGAAGGTGTAATCC;

36B4d CCCATTCTATCATCAACGGGTACAA [2].

Each qPCR was carried out in 384-well format which was vertically segmented in two parts: one for the telomeres (T) and one for the housekeeping gene 36B4 (S). Each 384-well plate contained the standard DNA, a quality control (commercially available DNA-Human Genomic DNA, Roche) and a non template control (NTC) in quadruplicate. All sample transfers and dilution steps were performed with a Tecan robotic workstation. Relative qPCR was carried out on an Applied Biosystems Taqman Fast Real-Time PCR 7900HT System. The thermal cycling began with the initial polymerase activation step (10 min at 95°C) and was followed by 40 cycles of 95°C for 15 s, 60°C for 1 min. A melting curve analysis to verify the specificity and identity of the products was performed.

The relative quantities were determined by the efficiency correction method [3], which does not need calibration curves and includes the individual real-time PCR efficiencies. This mathematical model calculates the ratio of a target gene (telomere) from the efficiencies and Ct-values of an experimental sample versus a reference gene (housekeeping gene) referring to a standard. To calculate PCR efficiencies of both the reference gene and the target gene PCR raw data were imported into the program LinRegPCR (version 12.5.0) [4]. Efficiencies were computed for all replicates of each sample. To check PCR data for outliers, the coefficients of variation (intra-assay CVs) were assessed for the Ct-values and the efficiency-values of quadruplicates in each gene. All single outlying values (CV>5%) were removed from further analyses. In the case of the housekeeping gene 36B4, about 2.2% in CRISIS and 5% in MMKD and in case of telomere, about 1.3% in CRISIS and 5% in MMKD were removed. For further mathematical analysis, the mean value of all efficiency-values of each gene on each plate and the mean Ct-value of the four replicates for each gene and each sample were used. Relative T/S-ratios reflect relative telomere length differences.

$$\text{Relative T}/S - \text{Ratio} = \frac{\text{eff (tel,sample)}^{\text{Ct (tel,sample)}}}{\text{eff (ref,sample)}^{\text{Ct (ref,sample)}}} \div \frac{\text{eff (tel,standard)}^{\text{Ct (tel,standard)}}}{\text{eff (ref,standard)}^{\text{Ct (ref,standard)}}}$$

To test the reproducibility of RTL measurement, the inter-assay CV of T/S-ratios was calculated according to the following formula:

$$\frac{\sqrt[2]{\frac{\sum_{i=1}^{n}(x_i - y_i)^2}{2n}}}{(\bar{X}+\bar{Y})\div 2}.$$

About 5% of all samples in both studies were analyzed in duplicate. Duplicate samples were never positioned on the same plate or at the same plate position. They were taken from different original DNA plates. Inter-assay CV of T/S-ratios of duplicates was 7.8% in CRISIS. As second RTL measurement quality control, we analyzed the relative telomere length of a commercially available DNA, which was positioned on each 384-well plate (a total of 24 plates). Inter-assay CV of T/S-ratios of this 24 times analyzed sample was 3.4%. In case of MMKD, inter-assay CV of T/S-ratios of duplicates was 12% and of quality control 6.9% (6 plates).

T/S-ratio values of CRISIS and MMKD are not directly comparable as different DNA extraction methods were used but can be interpreted in the same manner within both studies.

Several methods of TL measurement have been published so far. Compared to the Southern blot method, the quantitative polymerase chain reaction (qPCR) requires far less amount of DNA and is a high-throughput method. It is therefore widely used for large epidemiological studies. The qPCR assay provides the ratio of the telomere (T) and the housekeeping gene 36B4 (S) that should be proportional to the RTL of an individual. The comparison between these two methods has been published previously [5].

Statistical Analysis

Line plot

Marginal mean values and 95% CI of relative telomere length (RTL) (age- and sex-adjusted) were derived by general linear regression models. In a next step, differences in marginal mean values were compared over chronic kidney disease (CKD) stages defined by the Kidney Disease Outcomes Quality Initiative (KDOQI) guidelines.

Proportional hazards assumption

All calculated Cox regression analyses in the MMKD Study did not depart from the proportional hazards assumption. In the CRISIS Study, in the total group and in non-smokers and non-diabetics, the fully adjusted Cox regression Model 3 did depart from the proportional hazards (PH) assumption. As the deviation from the PH assumption was mainly due to GFR, we included a covariate in the respective models that accounts for the time-dependency of GFR. Thus, the HRs shown for relative telomere length are adjusted for this time effect (see Table 2).

Sensitivity analysis

To exclude that the association of RTL with progression of CKD in active smokers and patients with diabetes is confounded by angiotensin-converting-enzyme inhibitors (ACEi) (available in CRISIS and the MMKD Study) we adjusted the meta-analysis (Cox regression model 3) in active smokers additionally for ACEi. The effect for RTL was nearly unchanged (HR=1.40 [95%CI 1.12-1.75]; p=0.004). The same holds true for the effect of RTL in diabetics in CRISIS (HR=1.17 [95%CI 1.00-1.37]; p=0.05) and when adjusting for angiotensin receptor blockers (ARBs) (available in CRISIS only): HR=1.22 [95%CI 1.03-1.46]; p=0.02. These analyses revealed no difference in risk estimates of RTL.

Investigation of the yearly GFR change in the CRISIS Study

The first and the last eGFR calculation of the CRISIS study were used to calculate an average yearly GFR decline. A non-linear P-spline was used to check for linearity of yearly GFR change in a general linear regression analysis on RTL in active smokers. Yearly GFR change was inverse-normal transformed due to its skewed distribution. This analysis was adjusted for age, sex, proteinuria and GFR at baseline. As can be seen from Supplementary Figure 1 linearity was not present and we therefore performed the analysis using tertiles.

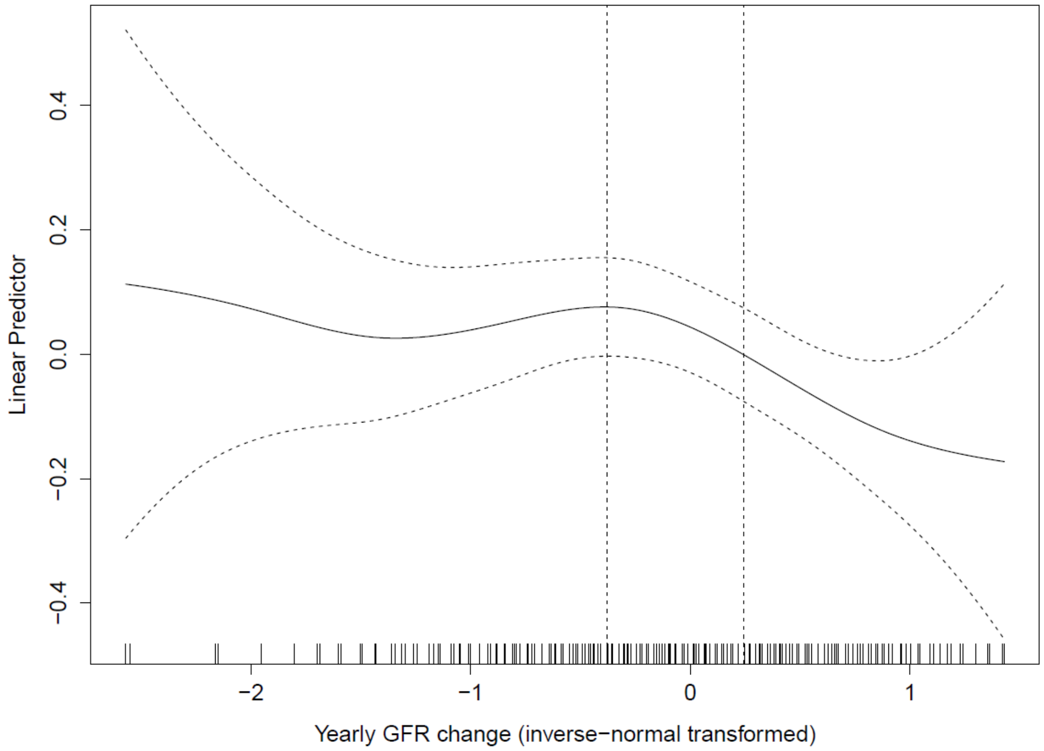

Supplementary Figure 1: *Non-linear P-spline to check for linearity of yearly GFR change in a general linear regression analysis on RTL in active smokers (for explanation see text). Vertical lines represent the borders of the tertiles.*

References

1. Cawthon, R. M. Telomere measurement by quantitative PCR. *Nucleic Acids Res.* **30**, e47 (2002).

2. Willeit, P. *et al.* Cellular aging reflected by leukocyte telomere length predicts advanced atherosclerosis and cardiovascular disease risk. *Arterioscler. Thromb. Vasc. Biol.* **30**, 1649-1656 (2010).

3. Pfaffl, M. W. A new mathematical model for relative quantification in real-time RT-PCR. *Nucleic Acids Res.* **29**, e45 (2001).

4. Ruijter, J. M. *et al.* Amplification efficiency: linking baseline and bias in the analysis of quantitative PCR data. *Nucleic Acids Res.* **37**, e45 (2009).

5. Ehrlenbach, S. *et al.* Influences on the reduction of relative telomere length over ten years in the population-based Bruneck Study: introduction of a well-controlled high-throughput assay. *Int. J. Epidemiol.* **38**, 1725-1734 (2009).
